# Effects of age and APOE ϵ4 genotype on the relationship between pulse pressure and executive function in older adults without Dementia

**DOI:** 10.1017/S1355617726102008

**Published:** 2026-05-13

**Authors:** Melanie L. Quiring, Lauren Edwards, Katherine J. Bangen, Emily H. Ho, Cynthia M. Carlsson, Douglas Galasko, Sandra Weintraub, Richard Gershon, David P. Salmon

**Affiliations:** 1 Gerontology, University of Southern California, USA; 2 Psychology Joint Doctoral Program, University of California, San Diego/San Diego State University, USA; 3 Psychiatry, University of California, San Diego, USA; 4 Psychology and Research Services, VA San Diego Healthcare System, San Diego, USA; 5 Psychiatry and Behavioral Sciences and Mesulam Institute for Cognitive Neurology and Alzheimer's Disease, Northwestern University Feinberg School of Medicine, USA; 6 University of Wisconsin-Madison School of Medicine and Public Health, USA; 7 Neurosciences, University of California, San Diego, USA

**Keywords:** Aging, arterial stiffness, blood pressure, cognition, executive function, vascular disease

## Abstract

**Objectives:**

Pulse pressure (PP) calculated as systolic minus diastolic blood pressure is a surrogate measure of arterial stiffness that may affect executive function; however, this relationship could be moderated by age and genetic risk for Alzheimer’s disease (AD). We therefore examined relationships among PP, age, AD risk (i.e., APOE genotype) and executive function measured by the NIH Toolbox Cognition Battery (NIHTB-CB) in older adults.

**Methods:**

PP was determined in 216 older adults without dementia (mean age: 77.5 ± 7.9 years, education: 16.8 ± 2.4 years, 55% women, 34.8% APOE ϵ4+) who were tested with the NIHTB-CB as part of the Advancing Reliable Measurement of Alzheimer’s Disease and Cognitive Aging (ARMADA) study.

**Results:**

Multiple linear regression revealed PP × Age × APOE genotype interaction effects for List Sorting Working Memory (*β* = 0.04; *p* = .007) and Picture Sequence Memory (*β* = 0.04; *p* = .006); higher PP was associated with worse scores in younger APOE ϵ4+ older adults (same pattern for fluid and total cognition composite scores). Higher PP was associated with lower Picture Vocabulary scores in ApoE ϵ4+ (PP X APOE interaction: *β* = −0.19; *p* = .022). Higher PP was associated with lower Flanker Inhibitory Control scores (*β* = −0.13; *p* = .005) across all participants.

**Conclusions:**

Arterial stiffness measured by PP in older adults is associated with worse performance on NIHTB-CB tests of executive function, working memory, and episodic sequence memory, particularly in younger APOE ϵ4 carriers. Arterial stiffness and AD risk may work synergistically in an age dependent manner to adversely affect cognition.

## Statement of Research Significance

**Research Question(s) or Topic(s):** Do age-related vascular changes such as increased arterial stiffness impact cognitive function and is this impact modified by presence of risk factors associated with Alzheimer’s disease? **Main Findings:** Higher pulse pressure, an easily obtained surrogate of increased arterial stiffness, was associated with worse performance by older adults on various tests of executive function and working memory contained in the National Institute of Health Toolbox Cognition Battery. In some cases, the association was strongest in those with a genetic risk for Alzheimer’s disease (i.e., Apolipoprotein ϵ4 allele) or less aging. **Study Contributions:** This study showed that arterial stiffness and Alzheimer’s disease risk can work synergistically in an age-dependent manner to adversely affect aspects of executive function in non-demented older adults.

## Introduction

Pulse pressure (PP) determined by the difference between systolic and diastolic blood pressure (BP) is an indirect measure of arterial stiffness (Safar et al., 2003). Increased arterial stiffness places pulsatile stress on capillaries in the brain which may contribute to microvascular damage (Reas et al., 2021), neuronal injury (Mitchell, 2008; Parada et al., 2023), and white matter lesions (Nation et al., 2010). Elevated PP in older adults is associated with cognitive decline (King et al., 2023; Nation et al., 2015; Waldstein et al., 2008) and increased risk of dementia (Qiu et al., 2003), particularly in those carrying the apolipoprotein E (APOE) ϵ4 allele (Bangen et al., 2021; Nation et al., 2016), the strongest genetic risk factor for sporadic Alzheimer’s disease (AD; Van Cauwenberghe et al., 2016). Pulse pressure and other measures of arterial stiffness correlate with AD biomarkers in cerebrospinal fluid (CSF) of older adults without dementia (Hughes et al., 2013; Moore et al., 2021; Nation et al., 2013, 2015; Shi et al., 2020) suggesting that arterial stiffness may indirectly contribute to cognitive decline by exacerbating early accumulation of AD pathology (Hughes et al., 2018; Nation et al., 2013, 2015; Parada et al., 2023). The specific nature of this relationship may be moderated by age, as higher PP is associated with lower CSF amyloid β (Aβ) (which is indicative of greater Aβ-positive plaque accumulation in the brain) in non-demented adults over 80 years of age and with higher phosphorylated tau (p-tau) in those younger than 80 years (Nation et al., 2015).

A number of studies have shown that PP and other measures of arterial stiffness (e.g., pulse wave velocity) are negatively associated with performance on standard neuropsychological tests of attention and executive function in older adults (Bangen et al., 2021; Kritz-Silverstein et al., 2017; Mitchell et al., 2011; Nation et al., 2016; Zang et al., 2021; Zhong et al., 2014), although, in some cases, effects were strongest or only observed in APOE ϵ4 carriers (Bangen et al., 2021; Cambronero et al., 2018; Parada et al., 2023). These findings are consistent with the known association between arterial stiffness and subcortical ischemic vascular pathology (Lamar et al., 2010; Miyagi et al., 2023; van Sloten et al., 2015) that most often affects subcortical-frontal circuits involved in regulating directed attention, inhibitory control, response choice, planning and sequencing, and working memory (Shallice & Burgess, 1991; Wetzel & Kramer, 2008). Other studies have shown that elevated PP and arterial stiffness are associated with decline on some standard neuropsychological tests of memory (Cambronero et al., 2018; McFall et al., 2015; Mitchell et al., 2011; Waldstein et al., 2008; Zang et al., 2021), language (Nation et al., 2010; Zang et al., 2021), or visuospatial abilities (only in APOE ϵ4 carriers; Nation et al., 2016); however, tests used in these studies had high retrieval demands (e.g., free recall of episodic or semantic memory) or planning constraints (e.g., drawing complex figures) that may have engaged executive functions in addition to the cognitive process they targeted.

Given that PP may interact with age or APOE genotype to affect cognition, we examined relationships among PP, age, AD risk determined by APOE genotype, and cognition in non-demented older adults. Cognition was assessed with the Cognition Battery of the National Institutes of Health (NIH) Toolbox for Assessment of Neurological and Behavioral Function (NIHTB-CB; Gershon et al., 2013) in non-demented older adults in the Advancing Reliable Measurement in Alzheimer’s Disease and Cognitive Aging (ARMADA) study (Weintraub et al., 2022). The NIHTB-CB has seven brief iPad-based computer-adaptive tests that measure executive function (i.e., set-shifting, inhibitory control), attention, processing speed, working memory, episodic memory, and language. In addition to standardized scores for each cognitive test, composite scores for fluid cognition, crystallized cognition, and total cognitive function are generated. The NIHTB-CB may be effective for assessing executive functions and other executive-supported cognitive processes that are vulnerable to fronto-subcortical damage that may occur with arterial stiffening in older adults with increased risk of AD due to an APOE ϵ4 genotype. We predicted that elevated PP would be associated with worse cognitive performance on NIHTB-CB tests that engage executive functions and fluid cognition but not with those that engage crystallized cognition (e.g., language, reading), and that the relationship would be moderated by age and AD risk.

## Method

### Participants

ARMADA participants were recruited from nine Alzheimer’s Disease Research Centers (ADRCs) or ADRC-affiliated research sites across the United States. The overall ARMADA cohort includes 462 older adults who were classified as cognitively normal (CN) or diagnosed with amnestic MCI (Albert et al., 2011) or AD dementia (McKhann et al., 2011) based on a clinical evaluation by the participating ADRC within 4 months of NIH-TB testing. The racial composition of the ARMADA cohort is proportionate to the US population. Special efforts were made to include individuals over the age of 85 (Mather et al., 2024). General exclusion criteria were any acute neurological disorder that could cause cognitive impairment or a history of major psychiatric disorder or substance abuse. In addition, serious medical illness was an exclusion for CN participants (inclusion/exclusion criteria and recruitment methods are detailed in Weintraub et al., 2022). The research was completed in accordance with the Helsinki Declaration and all participants provided informed consent following the local Institutional Review Board at each enrollment site (i.e., Northwestern University [lead site], University of Michigan, University of Wisconsin-Madison, Mayo Clinic-Jacksonville, University of Pittsburgh, Emory University, University of California-San Diego, Columbia University, Massachusetts General Hospital).

The present study included the 216 ARMADA participants who had a BP measurement and had received a diagnosis of CN (*n* = 136) or amnestic MCI (*n* = 80) at the ADRC clinical evaluation proximate to NIH-TB testing. The sample averaged 77.5 years of age (s.d. = 7.9, range = 64–99), 16.8 years of education (s.d. = 2.4; range = 8–21), and was 55.1% female participants, 13.9% Black participants, and 2.3% Latino or Hispanic participants. Approximately 33% of participants had at least one APOE ϵ4 allele (APOE ϵ4+). The average score on the Montreal Cognitive Assessment (MoCA) was 24.7 (s.d. = 3.5; range = 12–30) and the average Clinical Dementia Rating (CDR) sum of boxes was 0.7 (s.d. = 1.1; range = 0–9). Although some MoCA and CDR sum of boxes scores fell below typical cut-off scores for impairment, the classification as CN was based on results and interpretation of the full ADRC clinical evaluation and not any single measure.

### Procedure

Group membership (CN for age, mild cognitive impairment) had been determined at each ADRC or ADRC-affiliated study based on data from the uniform set of assessment procedures developed for the National Alzheimer’s Coordinating Center Uniform Data Set (UDS, version 3.0; Besser et al., 2018) and research diagnostic criteria. The UDS used standard methods to obtain medical history, list of current medications, physical examination, neurologic examination, neuropsychological testing, and assessment of daily function (Morris et al., 2006; Weintraub et al., 2018). APOE genotype was determined, and participants were classified as APOE ϵ4+ (ϵ3/ϵ4, ϵ4/ϵ4, or ϵ2/ϵ4) or APOE ϵ4- (ϵ2/ϵ2, ϵ2/ϵ3 or ϵ3/ϵ3). Brachial artery BP while seated was measured in a standardized manner (Muntner et al., 2019) as part of the physical examination. Pulse pressure was calculated by subtracting diastolic BP from systolic BP. Potential vascular risk factors including diabetes mellitus, atrial fibrillation, and hypercholesterolemia were determined based on review of medical history. Body mass index (BMI) was calculated as the individual’s weight in kilograms divided by the square of their height in meters. Although not a validated composite, a simple vascular risk score that ranged from 0 to 4 was derived by summing the number vascular risk factors (i.e., diabetes, hypercholesterolemia, or atrial fibrillation, and BMI ≥ 30) that were present.

### NIH toolbox cognition battery (NIHTB-CB)

The NIHTB Cognition Battery (NIHTB-CB) Version 2 was administered via iPad with a trained examiner present to ensure that participants understood the requirements of the tests. The NIHTB-CB consists of seven separate tests that measure language, executive function, memory, and processing speed (described below). Composite measures of fluid cognition, crystallized cognition, and total cognition can also be derived. Instructions for each task are presented as text and audio and preceded by practice trials to familiarize the participant with the task. An incorrect response on a practice trial causes an audio prompt to select the correct response. A required number of practice trials must be completed correctly in order to continue to the test. Each test takes 3–7 minutes to complete, and the total test time is 20–30 minutes. All participants were tested in English for the present study. The NIHTB Scoring and Interpretation Guide provides detailed scoring methodology for each test (NIH, 2016).

#### Dimensional change card sort test

This measure of executive function assesses cognitive flexibility and attention. On each trial, two target pictures that vary in shape and color (e.g., a blue ball and a yellow truck) are presented simultaneously on the iPad screen. While they remain present, a dimensional cue word (e.g., “color”) is presented for 1.5 seconds, followed by a test picture (e.g., a blue truck) and the participant must select the target (by touching it) that matches the test stimulus on the cued dimension (e.g., in this case, the blue ball). The target stimuli, test stimulus, and dimensional cue word vary across trials. A total of 40 trials are presented. Participants are instructed to answer as fast as they can without making mistakes. Trial by trial accuracy and reaction time (RT) are measured. Score is based on a combination of accuracy and RT.

#### Flanker inhibitory control and attention test

This measure of executive function assesses inhibitory control and attention. On each trial, a row of five arrows is presented on the iPad screen. On some trials, the middle arrow points in the same direction as the other flanking arrows (congruent), while on other trials, the middle arrow points in the opposite direction (incongruent) from the other arrows. Participants are instructed to select the direction of the center arrow as fast as they can without making mistakes. A total of 20 trials are presented with congruent and incongruent trials in an intermixed order that appears random to the participant. Participants must focus on the direction of the center arrow while inhibiting attention to the direction of flanking arrows which can either be the same as the central arrow (congruent trials) or opposite (incongruent). For each trial, accuracy and RT are measured. Scoring is based on a combination of accuracy across all trials and the median reaction time for incongruent trials.

#### Pattern comparison processing speed test

On each trial of this measure of cognitive processing speed, two simple pictures are presented side-by-side and the participant must decide and respond, as quickly as possible, whether the pictures are the same (“yes”) or not (“no”). Once the participant selects “yes” or “no,” a new pair of pictures is displayed. Trials with same or different pictures are randomly intermixed across trials. Testing continues for 90 seconds. Trial by trial accuracy and RT are measured. The raw score is the number of correct responses made in 90 seconds (with a maximum score of 130).

#### List sorting working memory test

This measure of working memory requires immediate recall of a list of items in an appropriate sequence. A series of pictures of animals (or food items) is presented one-at-a-time in a random order on the iPad screen. The name of each item is presented auditorily and as text simultaneously with the picture. After completion of list presentation, the participant must repeat the names of the items back in order of size of the item (i.e., smallest to largest) (1-List condition). In a second condition, the task is repeated with a series of pictures that contains both animals and food items intermixed, and the participant is asked to recall the food items in order of size and then the animals in order of size (2-List condition). Scoring is based on the total number of correctly recalled items on 1-List and 2-List trials.

#### Picture sequence memory test

This measure of episodic memory assesses memory for a sequence of events presented as pictures of events described in an audio recording. On each of two trials, 15 pictures of events that are components related to a theme (e.g., camping) are presented one at a time on the iPad screen. The event in the picture is also described auditorily. The trial starts with the presentation of the first picture/event in the center of the screen and its description (e.g., “When you go camping, first you set up the tent”). Once described, the picture remains on the screen and moves to its fixed position in the sequence (bordering the center of the screen). The next picture then immediately appears in the center of the screen with its audio description (e.g., “then, you go for a hike”) and then moves to the next fixed position in the sequence. This continues until all 15 pictures are displayed in the correct sequence for the participant to observe. The pictures are then moved to the center of the screen in a random spatial array and the participant must move each picture from the center of the screen to its correct location, replicating the learned sequence. Scoring is based on the number of correctly produced adjacent pairs. Participants complete two 15-item trials, each with different themes and stimuli.

#### Fluid cognition composite

The Fluid Cognition Composite score is a global measure of executive function, memory, and processing speed that is computed by averaging standard scores from Dimensional Change Card Sort, Flanker, Pattern Comparison, List Sorting, and Picture Sequence Memory, then deriving standard scores based on the new distribution.

#### Oral reading recognition test

This measure of language ability assesses reading decoding skill for single letters and words, including some low-frequency words and words with irregular orthography. On each trial, a word is presented on the iPad screen and the participant is asked to read and pronounce the word as accurately as possible. The test uses an adaptive procedure based on Item Response Theory to determine reading level.

#### Picture vocabulary test

This measure of language ability assesses general vocabulary knowledge. On each trial, four photographs are displayed on the iPad screen and a word that matches one of the pictures is auditorily presented. Participants are asked to select (by touching) the photograph that most closely matches the meaning of the word. The distractor photographs on each trial are semantically similar, visually similar, or antithetical to the target word, or a common misconception of the target word. The test uses an adaptive procedure based on Item Response Theory to determine vocabulary level.

#### Crystallized cognition composite

The Crystallized Cognition Composite score is a measure of language abilities and verbal cognition computed by averaging standard scores from Reading and Picture Vocabulary, then deriving standard scores based on the new distribution.

#### Cognitive function composite

The Cognitive Function Composite score is a measure of overall cognitive ability derived as the average of the Crystallized and Fluid Cognition Composite scores.

### Statistical Analyses

Separate multiple linear regression models were used to examine the relationship between PP and each of the ten NIHTB-CB measures. Each model included as independent predictors PP (grand-mean centered), age (grand-mean centered), APOE ϵ4 genotype (APOE ϵ4- vs. APOE ϵ4+), years of education, sex, vascular risk score (ranging from 0–4), current use of antihypertensive medications (yes vs. no), two-way interaction terms PP X Age and PP X APOE genotype, and the three-way interaction term PP X Age X APOE genotype. Age was modeled continuously in all inferential analyses and categorical age groups are used solely for illustrative purposes. The simplified vascular risk score was included in these analyses, rather than the individual vascular covariates, to minimize model complexity and collinearity with PP. The analyses used NIHTB-CB uncorrected standard scores generated automatically by the scoring program that compares raw scores to the entire NIHTB normative sample (NIH, 2016). Higher scores indicate better performance. Simple univariate relationships between PP and demographic and clinical characteristics of the sample were made with Pearson product-moment correlation for continuous variables (e.g., age, education, MoCA score) and with independent sample t-tests for dichotomous variables (e.g., sex, APOE genotype). Statistical significance threshold (alpha) was set at *p* < .05. False discovery rate (FDR) correction (Benjamini-Hochberg) was applied for the tests of three-way interactions (PP X APOE X Age) for each of the 10 cognitive measures, with *q* < 0.05 required for significance. FDR was limited to these tests since they were the primary tests of interest and other main effects and two-way interactions were more exploratory. Analyses were performed using Statistical Package for Social Sciences Version 28 and R for Windows Version 4.3.2.

## Results

The average systolic BP was 137.2 (s.d. = 18.6, range = 96–200), average diastolic BP was 77.3 (s.d. = 9.7, range = 55–106), and average PP was 59.9 (s.d. = 16.8, 11–114). Fifteen of the 216 participants reported a history of atrial fibrillation (6.9%), 29 a history of diabetes (13.4%), 130 a history of hypercholesterolemia (60.2%), and 128 the use of antihypertensive medication (59.8%). BMI was ≥30 for 43 participants (20.1%). The average vascular risk score was 1.0 (s.d. = 0.9, range = 0–4). Participants who had been clinically diagnosed with MCI did not differ from CN participants in average PP, systolic or diastolic BP, vascular risk factor score, use of antihypertensive medication, age, or education, although they had a higher percentage of men and APOE ϵ4 carriers (Supplementary Table 1). Pulse pressure was positively associated with age (*r* = 0.35; *p* < .05), negatively associated with MoCA score (r = −0.15; *p* < .05), and not significantly associated with vascular risk score (*r* = −0.08; *p* > .05). Pulse pressure was higher in men (mean = 62.11, s.d. = 17.86) than in women (mean = 57.23, s.d. = 15.00; *t* (214) = 2.15, *p* = .033, Cohen’s *d* = 0.29, 95% CI [0.02, 0.56]) but did not differ in those who were APOE ϵ4- (mean = 59.94, s.d. = 17.02) or ϵ4+ (mean = 59.58, s.d. = 15.99; *t* (206) = 0.14, *p* = .890, Cohen’s *d* = 0.02, 95% CI [−0.26, 0.31]). Uncorrected standard scores achieved on each NIHTB-CB test and composite are shown in Table 1.

**Table 1.** Mean (and standard deviation) uncorrected score achieved on each NIHTB-CB test or composite by the 216 non-demented ARMADA participants

NIHTB-CB cognitive test/composite	Mean score (s.d.)
Cognitive function composite	99.6 (9.6)
Fluid cognition composite	85.9 (12.3)
Domain dimensional change card sort test	96.8 (10.3)
Flanker inhibitory control and attention test	90.8 (8.6)
List sorting working memory test	91.2 (14.0)
Pattern comparison processing speed test	81.5 (14.7)
Picture sequence memory test	92.0 (12.9)
Crystallized cognition composite	114.3 (7.1)
Oral reading recognition test	110.9 (5.4)
Picture vocabulary test	116.5 (9.1)

**Figure 1.**
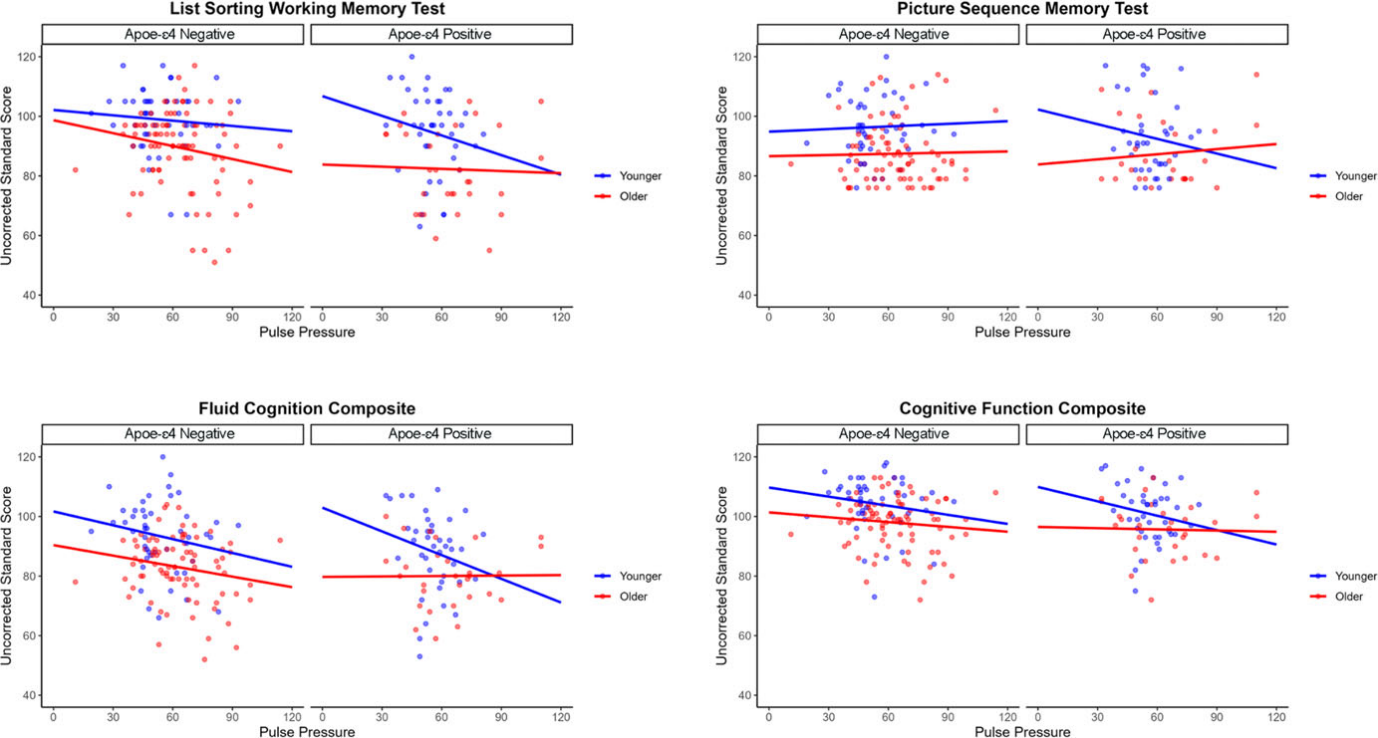
Significant PP X age X APOE interactions showed associations of pulse pressure with worse scores on the list sorting working memory test, picture sequence memory test, fluid cognition composite, and cognitive function composite were strongest for younger APOE ϵ4 positive participants.

The full results of the multiple linear regression models examining the relationship between PP and each of the ten NIHTB-CB measures are shown in Table 2. There were significant PP X Age X APOE interaction effects on the List Sorting Working Memory Test (*β* = 0.20; *p* = .007; FDR *q* = 0.032) and the Picture Sequence Memory Test (*β* = .20; *p* = .006; FDR *q* = 0.032). These interactions are depicted in Figure 1, where participants are stratified by APOE ϵ4 genotype and, for illustrative purposes, younger (<77 years old) or older (≥77 years old) age based on a median split. Higher PP was associated with lower scores on both tests, but the association was greater in those who were younger and APOE ϵ4+. Male sex (*β* = .20; *p* = .003) was also associated with lower Picture Sequence Memory Test scores. The List Sorting Working Memory and Picture Sequence Memory tests contribute heavily to the Fluid Cognition and Cognitive Function composite scores, so it is not surprising that there were also significant PP X Age X APOE interaction effects on both of these composites (Fluid Cognition Composite: *β* = 0.18; *p* = .011; FDR *q* = 0.037; Cognitive Function Composite: *β* = 0.17; *p* = .019; FDR *q* = 0.048). In both cases, higher PP was associated with lower scores and these associations were greater in those who were younger and APOE ϵ4+. Lower Fluid Cognition (*β* = 0.20; *p* = .003) and Cognitive Function (*β* = 0.29; *p* < .001) composite scores were associated with lower education.

**Table 2.** Results from multiple linear regression models examining the relationship between pulse pressure (PP), age, APOE genotype, other potentially associated variables, and scores on each of the ten NIHTB-CB measures

	Model			
	Est.	*SE*	*p*	β *(std.)*
Dimensional Change Card Sort Test				
Intercept	82.82	5.68		−0.02
PP	−0.06	0.06	.280	−0.12
**Age**	−0.37	0.12	**.002**	**−0.27**
**APOE**	−4.75	1.64	**.004**	**−0.22**
**Education**	0.95	0.31	**.002**	**0.21**
Sex	−0.24	1.47	.869	−0.01
Vascular risk score	0.05	0.86	.950	0.00
Antihypertensive medication use	−0.50	1.56	.751	−0.02
PP X Age	0.00	0.01	.808	0.07
PP X APOE	−0.03	0.10	.741	−0.02
Age X APOE	0.01	0.22	.948	0.00
PP X Age X APOE	0.02	0.01	.135	0.12
Flanker inhibitory control and attention test				
Intercept	75.53	4.67		−0.05
**PP**	−0.13	0.05	**.005**	**−0.23**
Age	−0.19	0.10	.061	−0.18
APOE	−1.64	1.34	.224	−0.09
**Education**	0.89	0.25	**<.001**	**0.24**
Sex	0.53	1.22	.660	0.03
Vascular risk score	−0.24	0.71	.735	−0.02
Antihypertensive medication use	0.92	1.29	.477	0.05
PP X Age	0.00	0.01	.473	0.14
PP X APOE	0.03	0.08	.662	0.03
Age X APOE	−0.02	0.18	.913	−0.01
PP X Age X APOE	0.01	0.01	.171	0.11
Pattern comparison processing speed test				
Intercept	71.69	8.12		−0.01
PP	−0.13	0.08	.108	−0.13
**Age**	−0.55	0.17	**.001**	**−0.29**
**APOE**	−5.15	2.34	**.029**	**−0.17**
Education	0.81	0.44	.064	0.13
Sex	0.06	2.11	.976	0.00
Vascular risk score	−1.62	1.23	.190	−0.09
Antihypertensive medication use	−1.55	2.24	.489	−0.05
PP X Age	0.00	0.01	.903	0.04
PP X APOE	0.03	0.14	.820	0.02
Age X APOE	0.05	0.31	.866	0.01
PP X Age X APOE	0.01	0.02	.413	0.06
List sorting working memory test				
Intercept	89.43	7.10		−0.02
PP	−0.01	0.07	.848	−0.03
**Age**	−0.62	0.15	**<.001**	**−0.41**
**APOE**	−9.76	2.04	**<.001**	**−0.34**
Education	0.51	0.38	.185	0.09
Sex	−1.18	1.85	.527	−0.04
Vascular risk score	−0.49	1.12	.659	−0.03
Antihypertensive medication use	−3.23	1.95	.099	−0.16
PP X Age	−0.01	0.01	.199	0.05
PP X APOE	−0.04	0.12	.739	−0.02
Age X APOE	−0.27	0.27	.316	−0.08
**PP X Age X APOE**	0.04	0.02	**.007**	**0.20**
Picture sequence memory test				
Intercept	82.99	6.90		0.01
PP	0.05	0.07	.464	0.00
**Age**	−0.65	0.14	**<.001**	**−0.33**
**APOE**	−4.25	1.98	**.033**	**−0.15**
Education	0.67	0.37	.074	0.12
**Sex**	5.33	1.78	**.003**	**0.20**
Vascular risk score	−1.58	1.05	.133	−0.10
Antihypertensive medication use	−3.29	1.88	.082	−0.12
PP X Age	−0.01	0.01	.321	0.07
PP X APOE	−0.15	0.12	.214	−0.09
Age X APOE	0.28	0.26	.276	0.08
**PP X Age X APOE**	0.04	0.02	**.006**	**0.20**
Fluid cognition composite				
Intercept	71.72	6.45		−0.02
PP	−0.07	0.06	.244	−0.11
**Age**	−0.67	0.14	**<.001**	**−0.41**
**APOE**	−6.47	1.85	**.001**	**−0.24**
**Education**	1.05	0.35	**.003**	**0.20**
Sex	1.17	1.67	.484	0.04
Vascular risk score	−1.00	1.02	.328	−0.07
Antihypertensive medication use	−2.07	1.75	.239	−0.08
PP X Age	0.00	0.01	.562	0.09
PP X APOE	−0.02	0.11	.832	−0.02
Age X APOE	0.05	0.24	.845	0.01
**PP X Age X APOE**	0.04	0.01	**.011**	**0.18**
Oral reading recognition test				
Intercept	99.04	2.81		−0.04
PP	0.01	0.03	.826	−0.04
Age	0.02	0.06	.701	0.02
APOE	−0.56	0.81	.486	−0.05
**Education**	0.78	0.15	**<.001**	**0.35**
Sex	−0.08	0.73	.910	−0.01
Vascular risk score	−0.74	0.43	.083	−0.12
Antihypertensive medication use	−0.30	0.77	.702	−0.03
PP X Age	0.00	0.00	.360	0.10
PP X APOE	−0.05	0.05	.278	−0.08
Age X APOE	−0.03	0.11	.811	−0.02
PP X Age X APOE	0.00	0.01	.695	0.03
Picture vocabulary test				
Intercept	95.70	4.77		0.05
PP	0.03	0.05	.470	−0.06
**Age**	−0.31	0.10	**.002**	**−0.20**
**APOE**	−2.83	1.37	**.041**	**−0.15**
**Education**	1.32	0.26	**<.001**	**0.34**
Sex	2.11	1.24	.090	0.11
**Vascular risk score**	−1.50	0.73	**.041**	**−0.14**
Antihypertensive medication use	1.07	1.31	.417	0.06
PP X Age	−0.01	0.01	.215	−0.05
**PP X APOE**	−0.19	0.08	**.022**	**−0.17**
Age X APOE	0.23	0.18	.204	0.10
PP X Age X APOE	0.01	0.01	.343	0.07
Crystallized cognition composite				
Intercept	96.75	3.69		0.02
PP	0.02	0.04	.578	−0.05
**Age**	−0.16	0.08	**.039**	**−0.14**
APOE	−1.87	1.06	.080	−0.12
**Education**	1.13	0.20	**<.001**	**0.38**
Sex	1.03	0.96	.285	0.07
**Vascular risk score**	−1.24	0.56	**.029**	**−0.15**
Antihypertensive medication use	0.45	1.02	.656	0.03
PP X Age	0.00	0.00	.627	0.00
PP X APOE	−0.12	0.06	.054	−0.14
Age X APOE	0.11	0.14	.454	0.06
PP X Age X APOE	0.01	0.01	.476	0.05
Cognitive function composite				
Intercept	82.03	5.00		−0.01
PP	−0.03	0.05	.506	−0.10
**Age**	−0.48	0.11	**<.001**	**−0.37**
**APOE**	−4.91	1.43	**.001**	**−0.24**
**Education**	1.22	0.27	**<.001**	**0.29**
Sex	1.29	1.30	.319	0.07
Vascular risk score	−1.27	0.79	.109	−0.11
Antihypertensive medication use	−1.02	1.36	.452	−0.05
PP X Age	0.00	0.01	.484	0.07
PP X APOE	−0.07	0.08	.416	−0.06
Age X APOE	0.08	0.19	.671	0.03
**PP X Age X APOE**	0.03	0.01	**.019**	**0.17**

*Note.* Unstandardized estimates (Est.), standard errors (SE), and standardized betas (β) are presented. PP = Pulse pressure, APOE = APOE ϵ4 carrier status (dummy-coded such that 0 = non-carrier and 1 = carrier), Education = Years of education. Antihypertensive medication use was dummy-coded such that 0 = not using medication and 1 = currently using medication. Sex was dummy-coded such that 0 = male and 1 = female. PP and Age were grand-mean centered.

**Figure 2.**
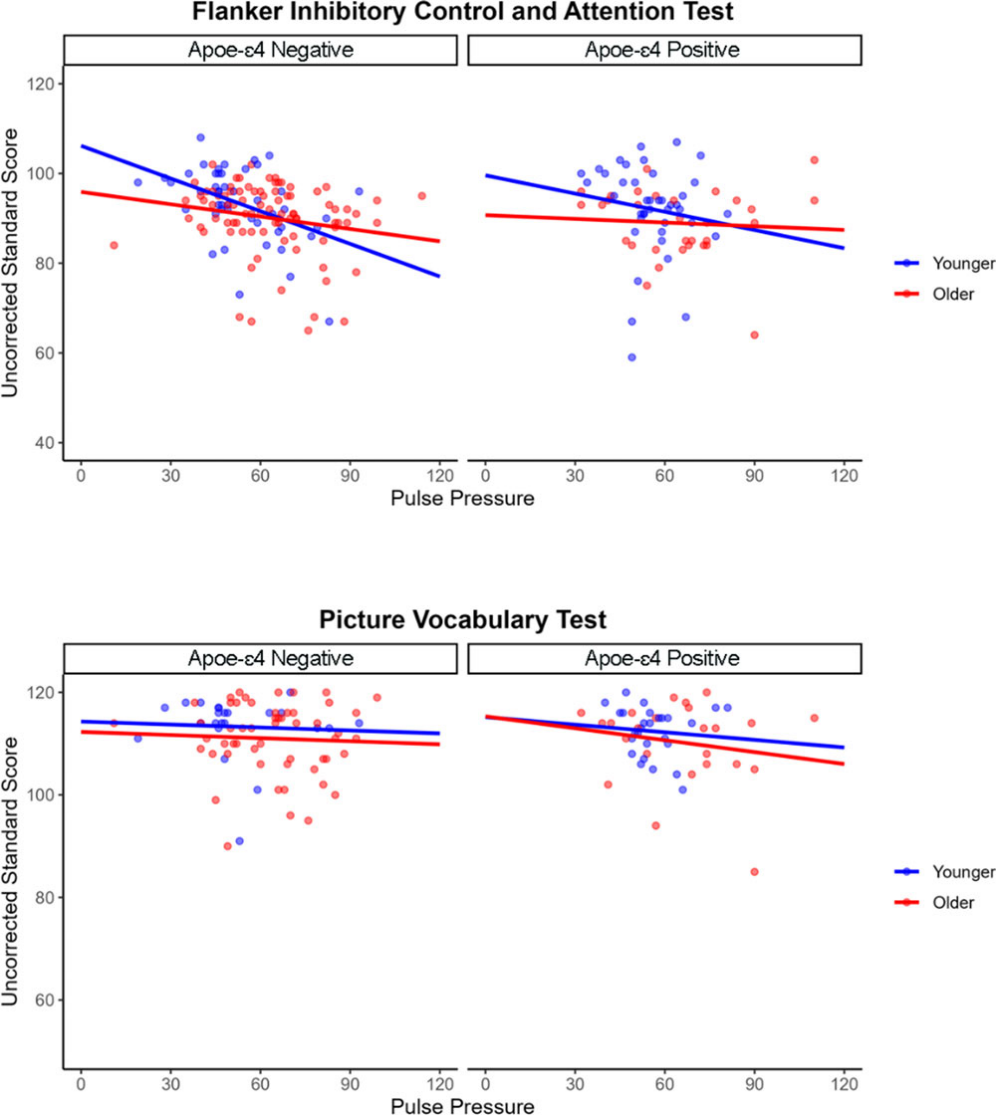
A main effect of PP indicated that pulse pressure was associated with worse scores on the Flanker inhibitory control and attention test across all participants. A significant PP X APOE interaction showed the association of pulse pressure with worse scores on the picture vocabulary test was strongest for APOE ϵ4 positive participants.

Higher PP was associated with lower scores on the Flanker Inhibitory Control and Attention Test independently of age or APOE genotype (*β* = −0.23; *p* = .005). The main effect of PP is illustrated in Figure 2a, where participants are stratified by APOE ϵ4 genotype and younger (<77 years old) or older (≥77 years old) age based on a median split. Lower education was also associated with lower Flanker Inhibitory Control and Attention Test scores (*β* = 0.24; *p* = .001). Pulse pressure was not associated with scores on the Dimensional Change Card Sort or Pattern Comparison Processing Speed tests. Lower scores on both tests were independently associated with older age (*β* = −0.27; *p* = .002 and *β* = −0.29; *p* = .001, respectively) and APOE ϵ4+ genotype (*β* = −0.22; *p* = .004 and *β* = −0.17; *p* = .029, respectively). Lower scores on the Dimensional Change Card Sort test were also associated with lower education (*β* = 0.21; *p* = .002).

Higher education was associated with higher scores on the Crystallized Cognition Composite (*β* = 0.38; *p* < .001) and on the Oral Reading Recognition Test (*β* = 0.35; *p* < .001) and Picture Vocabulary Test (*β* = 0.34; *p* < .001), the two crystallized cognition measures. Younger age was also associated with Higher Picture Vocabulary Test scores (*β* = −0.20; *p* = .002). Neither the Crystallized Cognition Composite nor the Oral Reading Recognition Test were associated with PP. Higher PP was more strongly associated with lower scores on the Picture Vocabulary Test in those who were APOE ϵ4+ than in those who were APOE ϵ4- (i.e., PP X APOE interaction effect: *β* = −0.17; *p* = .022). This interaction effect is illustrated in Figure 2b where participants are stratified by APOE ϵ4 genotype and younger (<77 years old) or older (≥77 years old) age based on a median split.

The same pattern of results was obtained when analyses were limited to CN participants, with significant PP X Age X APOE interaction effects on the Picture Sequence Memory Test (*β* = 0.17; *p* = .039) and the Cognitive Function Composite: *β* = 0.17; *p* = .042), and marginally significant PP X Age X APOE interaction effects on the List Sorting Working Memory Test (*β* = 0.14; *p* = .080) and the Fluid Cognition Composite (*β* = 0.15; *p* = .058). Other main effects and interaction effects remained significant, except when they involved APOE ϵ4+ genotype (see Supplemental Table S2).

## Discussion

Our results show that high PP, a surrogate measure of arterial stiffness, is associated with worse performance on NIHTB-CB measures of inhibitory control, working memory, episodic picture sequence memory, and language ability in older adults without dementia or history of stroke. These relationships are not explained by age, education, sex, aggregate vascular risk (or individual vascular risk factors in sensitivity analyses, see Supplemental Table S3), or use of antihypertensive medication; however, age and genetic risk for AD (i.e., APOE genotype) may moderate the relationship in some cases. Specifically, elevated PP is associated with worse performance on the NIHTB-CB measure of inhibitory control (i.e., a Flanker Task) independent of age or APOE ϵ4 genotype. It is also associated with NIHTB-CB measures of working memory and episodic memory, but these associations are most pronounced in younger APOE ϵ4 carriers. Modest association between PP and a measure of language ability (i.e., Picture Vocabulary) is stronger among APOE ϵ4 carriers than non-carriers regardless of age. These results suggest that arterial stiffness has adverse effects on various aspects of executive function in non-demented older adults, and that there may be a synergistic relationship between arterial stiffness and AD risk (i.e., APOE ϵ4 genotype), particularly in earlier stages of aging.

Our findings are consistent with previous studies that found PP and other measures of arterial stiffness (e.g., pulse wave velocity) primarily correlate with frontally mediated executive function test scores (Bangen et al., 2021; Cambronero et al., 2018; Kritz-Silverstein et al., 2017; Mitchell et al., 2011; Nation et al., 2016; Zang et al., 2021; Zhong et al., 2014). These studies largely relied on one or two standard neuropsychological tests that engage executive functions such as the Trail Making Test (Kritz-Silverstein et al., 2017; Nation et al., 2016; Waldstein et al., 2008; Zhong et al., 2014) or the DKEFS Tower Test (Cambronero et al., 2018), or executive function test composites (Bangen et al., 2021; Mitchell et al., 2011; Nation et al., 2010; Zang et al., 2021). We extend these results by showing that elevated PP is associated with poorer performance on various independent components of executive function including inhibitory control, working memory, and episodic memory for sequences. Furthermore, these associations may be moderated differently by age or APOE genotype depending on the specific executive function component.

Arterial stiffness is more strongly associated with lower performance on a broad range of cognitive skills in APOE ϵ4 carriers than non-carriers (Bangen et al., 2021; Bender & Raz, 2012; Cambronero et al., 2018; Nation et al., 2016). Similarly, our results show that elevated PP interacts with APOE ϵ4 genotype to lower working memory, episodic sequence memory, and some language abilities, either alone or as part of Fluid Cognition or overall Cognitive Function composite scores. This interactive effect may result from a direct role of APOE ϵ4 in vascular dysfunction or from an indirect role by increasing likelihood of AD. Evidence for a direct role is that APOE ϵ4 carriers appear to be more vulnerable than non-carriers to vascular insults caused by arterial stiffening (Bangen et al., 2021; Cambronero et al., 2018) and more likely to have blood-brain barrier breakdown (Bell et al., 2012) and Aβ accumulation in the walls of blood vessels (Castellano et al., 2011). Evidence for an indirect role is that a recent study showed that arterial stiffness measured by pulse wave velocity interacts with CSF AD biomarker positivity, but not APOE ϵ4 genotype, to predict decline in executive function and language abilities in non-demented older adults (Edwards et al., 2025). In addition, cognitive abilities prominently affected by AD such as episodic memory (Bangen et al., 2021) and semantic memory (i.e., language measures like the Picture Vocabulary test in the present study) are more strongly associated with arterial stiffness in APOE ϵ4 carriers than non-carriers. Even measures generally resilient to age and vascular changes such as the Picture Vocabulary tests can be affected by early AD changes that are more likely in those with an APOE ϵ4 genotype. Resilience to vascular changes may be reduced in the context of AD if it increases vulnerability of semantic networks or reduces brain/cognitive reserve. Inhibitory control, in contrast, may be affected by vascular effects alone since we found no interaction between PP and APOE ϵ4 genotype for this aspect of executive function.

Our results are also consistent with evidence that negative effects of elevated PP on executive functioning (Kritz-Silverstein et al., 2017), and neural damage caused by arterial stiffness (Reas et al., 2021), are more pronounced in adults under 80 years of age than in those over 80. This has been attributed to the possibility that higher systolic BP may help maintain adequate brain perfusion in very old adults (Kritz-Silverstein et al., 2017). In the present study age was modeled continuously, but the relationship between elevated PP and worse working memory or episodic sequence memory gradually strengthened with decreasing age, particularly in those who were APOE ϵ4 positive. Nation et al. (2015) found that PP is associated with CSF phosphorylated tau (P-tau) concentrations in adults less than 80 years of age, but with CSF amyloid concentration in adults 80 years of age or older. Thus, the relationship between elevated PP and poorer executive function we observed in those at risk for AD (i.e., APOE ϵ4 genotype) may be most evident in younger individuals since P-tau is a stronger predictor of cognitive decline than amyloid burden (Hanseeuw et al., 2019; Sperling et al., 2024). Adverse effects of elevated PP may also be less salient in very old age as the prevalence of other age-related pathologies increases (Nation et al., 2013).

Several limitations should be considered. While the NIHTB-CB assesses specific aspects of executive function that might be differentially affected by arterial stiffness, the measure of episodic memory, the Picture Sequence Memory Test, requires the ability to learn a spatial sequence of pictures over several trials, but with little delay needed to assess retention, and a significant demand on executive function needed to recreate sequences. Thus, it is difficult to separate PP’s association with executive function from its association with retentive episodic memory in the present study. Additional measures of delayed recall and recognition that are now available in the full NIHTB (e.g., Rey Auditory Verbal Learning Test) were not administered in the present study due to time limitations. A second limitation is the low availability of AD biomarkers (e.g., amyloid PET, CSF Aβ_1–42_, plasma p-Tau217) or neuroimaging data in this subsample of the ARMADA cohort (available for only 30% of the subsample) which restricts our ability to determine whether the cognitive effects associated with the APOE ϵ4 genotype are a function of vascular dysfunction or indirectly related to increased AD risk. Neuroimaging data might help identify underlying mechanisms (e.g., white matter hyperintensities, prefrontal cortical atrophy, reduce cerebral blood flow to prefrontal neocortical networks) driving the relationship between poorer executive function and arterial stiffness. A third limitation is that PP was derived from a single seated brachial BP measurement and other, more direct, measures of central arterial stiffness (e.g., pulse wave velocity) were not available. However, PP is a widely accepted and easily obtained surrogate measure of arterial stiffness that can be effectively translated into clinical practice. A fourth limitation is the cross-sectional design of the study which precludes strong inference about the causal relationship between increased PP and executive dysfunction. Finally, our study was limited to English speakers, with only 2% identifying as Latino or Hispanic, even though the overall ARMADA sample is racially representative of the US population and included a high proportion of very old and highly educated adults (Karpouzian-Rogers et al., 2023; Weintraub et al., 2022). These demographic limitations, due in part to BP not being measured at all sites, reduce the generalizability of our findings. Despite these limitations, our results indicate that elevated PP is associated with worse performance on NIHTB-CB tests that engage executive functions and fluid cognition, but not with those that engage crystallized cognition (e.g., language, reading), moderated by age and AD risk.

## Supporting information

10.1017/S1355617726102008.sm001 Quiring et al. supplementary material Quiring et al. supplementary material

## Supplementary material

The supplementary material for this article can be found at https://doi.org/10.1017/S1355617726102008.
