# Supplementary material for: Effects of age and APOE ϵ4 genotype on the relationship between pulse pressure and executive function in older adults without Dementia
Source: J Int Neuropsychol Soc. 2026 May 13:1–10. Online ahead of print. doi: 10.1017/S1355617726102008 (PMC13175078; doi:10.1017/S1355617726102008)
Supplement: Quiring et al. supplementary material [file S1355617726102008sup001.docx]

**Supplemental Table S1.** Participant Demographics and Clinical Characteristics by Clinical Diagnosis: Cognitively Normal (CN) vs. Mild Cognitive Impairment (MCI). Mean (SD) value or n (%) with each characteristic are shown. Results of group comparisons with independent sample t-tests or χ^2^ tests and p-value are also shown.

| **Variable** | **CN**  ***n* = 136** | **MCI**  ***n* = 80** | ***t* or χ^2^** | ***p*** |
| --- | --- | --- | --- | --- |
| Age (yrs.) | 77.8 (8.2) | 77.0 (7.3) | 0.74 | .461 |
| Education (yrs.) | 16.8 (2.3) | 16.8 (2.4) | 0.16 | .436 |
| Female (n, %) | 86 (63.2%) | 33 (41.3%) | 9.84 | .002 |
| APOE ɛ4+ (n, %) | 36 (27.1%) | 36 (48.6%) | 9.76 | .002 |
| MoCA | 26.3 (2.7) | 22.0 (3.1) | 10.67 | .001 |
| CDR Sum of Boxes | 0.2 (0.5) | 1.5 (1.4) | -7.75 | .001 |
| Vascular Risk Score | 1.6 (1.2) | 1.6 (1.1) | 0.12 | .904 |
| Antihypertensive Medication Use (n, %) | 81 (59.6) | 47 (58.8) | 0.01 | .920 |
| Systolic Blood Pressure | 137.4 (29.1) | 136.9 (17.8) | 0.21 | .832 |
| Diastolic Blood Pressure | 77.5 (9.7) | 77.0 (9.6) | 0.36 | .720 |
| Pulse Pressure | 59.9 (16.9) | 59.9 (16.7) | 0.03 | .978 |

*Note.* APOE genotype was not available for nine participants. MoCA = Montreal Cognitive Assessment, CDR = Clinical Dementia Rating Scale.

**Supplemental Table S2:** Results from multiple linear regression models examining the relationship between pulse pressure (PP), age, APOE genotype, other potentially associated variables, and scores on each of the ten NIHTB-CB measures, in the 136 Cognitively Normal (CN) participants.

## Dimensional Change Card Sort Test

| Term | b | SE | t | p | β | 95% CI |
| --- | --- | --- | --- | --- | --- | --- |
| (Intercept) | 94.041 | 5.431 |  |  |  |  |
| PP | -0.084 | 0.052 | -1.617 | 0.109 | -0.204 | [-0.187, 0.018] |
| **Age** | -0.318 | 0.108 | -2.934 | **0.004** | -0.260 | [-0.530, -0.105] |
| APOE | -2.185 | 1.744 | -1.253 | 0.213 | -0.113 | [-5.603, 1.232] |
| Education | 0.494 | 0.302 | 1.635 | 0.105 | 0.140 | [-0.098, 1.086] |
| Sex | -2.649 | 1.479 | -1.791 | 0.076 | -0.156 | [-5.548, 0.251] |
| Vascular Risk Score | -1.222 | 0.878 | -1.392 | 0.167 | -0.129 | [-2.943, 0.499] |
| Anti-htn Medication | 0.198 | 1.609 | 0.123 | 0.902 | 0.012 | [-2.956, 3.352] |
| PP X Age | 0.009 | 0.006 | 1.508 | 0.134 | 0.168 | [-0.003, 0.021] |
| PP X APOE | -0.070 | 0.102 | -0.687 | 0.494 | -0.063 | [-0.269, 0.129] |
| Age X APOE | 0.226 | 0.215 | 1.054 | 0.294 | 0.101 | [-0.194, 0.647] |
| PP X Age X APOE | 0.003 | 0.012 | 0.213 | 0.832 | 0.019 | [-0.021, 0.026] |

## Flanker Inhibitory Control and Attention Test

| Term | b | SE | t | p | β | 95% CI |
| --- | --- | --- | --- | --- | --- | --- |
| (Intercept) | 93.145 | 1.618 |  |  |  |  |
| **PP** | -0.153 | 0.048 | -3.168 | **0.002** | -0.305 | [-0.248, -0.058] |
| **Age** | -0.206 | 0.101 | -2.043 | **0.043** | -0.181 | [-0.403, -0.008] |
| APOE | 0.127 | 1.615 | 0.079 | 0.937 | 0.009 | [-3.038, 3.292] |
| Vascular Risk Score | -0.632 | 0.816 | -0.774 | 0.441 | -0.071 | [-2.232, 0.968] |
| Sex | -1.459 | 1.376 | -1.060 | 0.291 | -0.091 | [-4.155, 1.238] |
| **Education** | 0.610 | 0.280 | 2.180 | **0.031** | 0.184 | [0.061, 1.158] |
| Anti-htn Medication | 0.849 | 1.490 | 0.569 | 0.570 | 0.054 | [-2.072, 3.770] |
| PP X Age | 0.007 | 0.006 | 1.274 | 0.205 | 0.191 | [-0.004, 0.019] |
| PP X APOE | 0.033 | 0.094 | 0.354 | 0.724 | 0.036 | [-0.151, 0.218] |
| Age X APOE | 0.144 | 0.199 | 0.724 | 0.470 | 0.065 | [-0.246, 0.534] |
| PP X Age X APOE | 0.013 | 0.011 | 1.125 | 0.263 | 0.100 | [-0.009, 0.034] |

## Pattern Comparison Processing Speed Test

| Term | b | SE | t | p | β | 95% CI |
| --- | --- | --- | --- | --- | --- | --- |
| (Intercept) | 71.121 | 32.924 |  |  |  |  |
| PP | -0.110 | 0.093 | -1.185 | 0.239 | -0.204 | [-0.293, 0.072] |
| **Age** | -0.755 | 0.196 | -3.856 | **<.001** | -0.347 | [-1.139, -0.371] |
| APOE | -5.474 | 3.118 | -1.756 | 0.082 | -0.153 | [-11.585, 0.636] |
| Education | 0.172 | 0.293 | 0.586 | 0.559 | 0.048 | [-0.402, 0.746] |
| Sex | -4.850 | 2.650 | -1.831 | 0.070 | -0.154 | [-10.043, 0.343] |
| Vascular Risk Score | -2.285 | 1.561 | -1.463 | 0.146 | -0.130 | [-5.345, 0.776] |
| Anti-htn Medication | -0.495 | 2.886 | -0.172 | 0.864 | -0.016 | [-6.151, 5.160] |
| PP X Age | 0.007 | 0.011 | 0.607 | 0.545 | 0.110 | [-0.015, 0.028] |
| PP X APOE | -0.288 | 0.180 | -1.599 | 0.113 | -0.139 | [-0.642, 0.065] |
| Age X APOE | 0.434 | 0.384 | 1.131 | 0.260 | 0.103 | [-0.318, 1.186] |
| PP X Age X APOE | 0.020 | 0.022 | 0.921 | 0.359 | 0.081 | [-0.022, 0.062] |

## List Sorting Working Memory Test

| Term | b | SE | t | p | β | 95% CI |
| --- | --- | --- | --- | --- | --- | --- |
| (Intercept) | 55.925 | 22.691 |  |  |  |  |
| PP | -0.030 | 0.064 | -0.475 | 0.635 | -0.025 | [-0.155, 0.094] |
| **Age** | -0.643 | 0.135 | -4.749 | **<.001** | -0.564 | [-0.909, -0.378] |
| **APOE** | -6.279 | 2.131 | -2.946 | **0.004** | -0.256 | [-10.457, -2.102] |
| Education | 0.397 | 0.202 | 1.967 | 0.051 | 0.151 | [0.001, 0.792] |
| Sex | -3.485 | 1.820 | -1.915 | 0.058 | -0.151 | [-7.052, 0.081] |
| Vascular Risk Score | -0.939 | 1.131 | -0.830 | 0.408 | -0.070 | [-3.156, 1.278] |
| Anti-htn Medication | -0.277 | 1.975 | -0.140 | 0.889 | -0.012 | [-4.149, 3.595] |
| PP X Age | -0.002 | 0.008 | -0.308 | 0.759 | 0.060 | [-0.017, 0.012] |
| PP X APOE | 0.044 | 0.124 | 0.357 | 0.722 | 0.034 | [-0.198, 0.286] |
| Age X APOE | -0.455 | 0.264 | -1.724 | 0.087 | -0.153 | [-0.972, 0.062] |
| PP X Age X APOE | 0.026 | 0.015 | 1.763 | 0.080 | 0.144 | [-0.003, 0.055] |

## Picture Sequence Memory Test

| Term | b | SE | t | p | β | 95% CI |
| --- | --- | --- | --- | --- | --- | --- |
| (Intercept) | 87.283 | 8.005 |  |  |  |  |
| PP | 0.043 | 0.077 | 0.562 | 0.575 | 0.004 | [-0.108, 0.194] |
| **Age** | -0.724 | 0.162 | -4.458 | **<.001** | -0.384 | [-1.042, -0.406] |
| APOE | 0.521 | 2.566 | 0.203 | 0.840 | 0.021 | [-4.509, 5.550] |
| Education | 0.761 | 0.444 | 1.713 | 0.089 | 0.134 | [-0.110, 1.632] |
| Sex | 2.592 | 2.188 | 1.184 | 0.239 | 0.094 | [-1.697, 6.881] |
| **Vascular Risk Score** | -3.001 | 1.299 | -2.310 | **0.023** | -0.196 | [-5.548, -0.455] |
| Anti-htn Medication | -3.974 | 2.378 | -1.672 | 0.097 | -0.147 | [-8.634, 0.686] |
| PP X Age | -0.003 | 0.009 | -0.323 | 0.748 | 0.075 | [-0.021, 0.015] |
| PP X APOE | -0.148 | 0.149 | -0.988 | 0.325 | -0.081 | [-0.440, 0.145] |
| Age X APOE | 0.346 | 0.317 | 1.091 | 0.277 | 0.091 | [-0.275, 0.967] |
| **PP X Age X APOE** | 0.037 | 0.018 | 2.088 | **0.039** | 0.171 | [0.002, 0.072] |

## Fluid Cognition Composite

| Term | b | SE | t | p | β | 95% CI |
| --- | --- | --- | --- | --- | --- | --- |
| (Intercept) | 81.736 | 6.527 | 12.523 |  |  |  |
| PP | -0.091 | 0.063 | -1.447 | 0.151 | -0.192 | [-0.214, 0.032] |
| **Age** | -0.737 | 0.135 | -5.452 | **<.001** | -0.493 | [-1.002, -0.472] |
| APOE | -3.752 | 2.090 | -1.796 | 0.075 | -0.147 | [-7.848, 0.343] |
| **Education** | 0.772 | 0.362 | 2.132 | **0.035** | 0.162 | [0.062, 1.481] |
| Sex | -2.400 | 1.802 | -1.332 | 0.185 | -0.103 | [-5.931, 1.131] |
| **Vascular Risk Score** | -2.304 | 1.127 | -2.045 | **0.043** | -0.170 | [-4.513, -0.096] |
| Anti-htn Medication | -1.057 | 1.939 | -0.545 | 0.587 | -0.047 | [-4.858, 2.745] |
| PP X Age | 0.006 | 0.007 | 0.777 | 0.439 | 0.165 | [-0.009, 0.020] |
| PP X APOE | -0.132 | 0.122 | -1.081 | 0.282 | -0.089 | [-0.371, 0.107] |
| Age X APOE | 0.203 | 0.260 | 0.781 | 0.436 | 0.061 | [-0.307, 0.713] |
| PP X Age X APOE | 0.028 | 0.014 | 1.915 | 0.058 | 0.152 | [-0.001, 0.056] |

## Oral Reading Recognition Test

| Term | b | SE | t | p | β | 95% CI |
| --- | --- | --- | --- | --- | --- | --- |
| (Intercept) | 102.809 | 2.898 |  |  |  |  |
| PP | -0.007 | 0.028 | -0.251 | 0.802 | -0.053 | [-0.062, 0.048] |
| Age | 0.097 | 0.058 | 1.686 | 0.094 | 0.214 | [-0.016, 0.211] |
| APOE | -0.074 | 0.930 | -0.079 | 0.937 | -0.005 | [-1.898, 1.750] |
| **Education** | 0.532 | 0.161 | 3.299 | **0.001** | 0.289 | [0.216, 0.847] |
| Sex | 1.156 | 0.789 | 1.465 | 0.146 | 0.130 | [-0.391, 2.703] |
| Vascular Risk Score | -0.187 | 0.469 | -0.398 | 0.691 | -0.038 | [-1.105, 0.732] |
| Anti-htn Medication | -0.667 | 0.859 | -0.777 | 0.439 | -0.077 | [-2.350, 1.016] |
| PP X Age | 0.000 | 0.003 | 0.082 | 0.935 | 0.078 | [-0.006, 0.007] |
| PP X APOE | -0.028 | 0.054 | -0.507 | 0.613 | -0.043 | [-0.134, 0.079] |
| Age X APOE | 0.057 | 0.115 | 0.494 | 0.622 | 0.046 | [-0.168, 0.281] |
| PP X Age X APOE | 0.008 | 0.006 | 1.239 | 0.218 | 0.114 | [-0.005, 0.021] |

## Picture Vocabulary Test

| Term | b | SE | t | p | β | 95% CI |
| --- | --- | --- | --- | --- | --- | --- |
| (Intercept) | 102.228 | 5.614 |  |  |  |  |
| PP | 0.004 | 0.054 | 0.083 | 0.934 | -0.022 | [-0.101, 0.110] |
| **Age** | -0.227 | 0.112 | -2.028 | **0.045** | -0.151 | [-0.446, -0.008] |
| APOE | -1.770 | 1.802 | -0.982 | 0.328 | -0.089 | [-5.302, 1.762] |
| **Education** | 0.922 | 0.312 | 2.954 | **0.004** | 0.258 | [0.310, 1.534] |
| **Sex** | 3.769 | 1.529 | 2.465 | **0.015** | 0.219 | [0.773, 6.766] |
| Vascular Risk Score | -0.907 | 0.908 | -0.999 | 0.320 | -0.094 | [-2.685, 0.872] |
| Anti-htn Medication | 0.731 | 1.663 | 0.440 | 0.661 | 0.043 | [-2.529, 3.991] |
| PP X Age | -0.009 | 0.006 | -1.431 | 0.155 | -0.109 | [-0.022, 0.003] |
| PP X APOE | -0.048 | 0.105 | -0.453 | 0.652 | -0.040 | [-0.253, 0.158] |
| Age X APOE | 0.261 | 0.222 | 1.177 | 0.242 | 0.113 | [-0.174, 0.696] |
| PP X Age X APOE | 0.009 | 0.012 | 0.759 | 0.449 | 0.070 | [-0.015, 0.034] |

## Crystallized Cognition Composite

| Term | b | SE | t | p | β | 95% CI |
| --- | --- | --- | --- | --- | --- | --- |
| (Intercept) | 102.308 | 4.088 |  |  |  |  |
| PP | -0.003 | 0.039 | -0.086 | 0.932 | -0.036 | [-0.080, 0.074] |
| Age | -0.072 | 0.081 | -0.878 | 0.381 | -0.039 | [-0.231, 0.088] |
| APOE | -1.075 | 1.312 | -0.819 | 0.415 | -0.075 | [-3.647, 1.498] |
| **Education** | 0.789 | 0.227 | 3.471 | **<.001** | 0.304 | [0.343, 1.234] |
| **Sex** | 2.500 | 1.113 | 2.246 | **0.027** | 0.200 | [0.318, 4.683] |
| Vascular Risk Score | -0.633 | 0.661 | -0.958 | 0.340 | -0.091 | [-1.928, 0.662] |
| Anti-htn Medication | 0.003 | 1.211 | 0.003 | 0.998 | 0.000 | [-2.371, 2.377] |
| PP X Age | -0.005 | 0.005 | -1.017 | 0.311 | -0.053 | [-0.014, 0.004] |
| PP X APOE | -0.032 | 0.076 | -0.418 | 0.677 | -0.036 | [-0.182, 0.118] |
| Age X APOE | 0.154 | 0.161 | 0.951 | 0.343 | 0.091 | [-0.163, 0.470] |
| PP X Age X APOE | 0.009 | 0.009 | 0.970 | 0.334 | 0.090 | [-0.009, 0.027] |

## Cognitive Function Composite

| Term | b | SE | t | p | β | 95% CI |
| --- | --- | --- | --- | --- | --- | --- |
| (Intercept) | 90.001 | 4.862 |  |  |  |  |
| PP | -0.059 | 0.047 | -1.262 | 0.209 | -0.183 | [-0.151, 0.033] |
| **Age** | -0.467 | 0.101 | -4.641 | **<.001** | -0.409 | [-0.665, -0.270] |
| APOE | -2.896 | 1.557 | -1.860 | 0.065 | -0.158 | [-5.947, 0.155] |
| **Education** | 0.930 | 0.270 | 3.449 | **<.001** | 0.271 | [0.401, 1.458] |
| Sex | -0.039 | 1.342 | -0.029 | 0.977 | -0.002 | [-2.669, 2.592] |
| **Vascular Risk Score** | -1.785 | 0.839 | -2.127 | **0.036** | -0.183 | [-3.430, -0.140] |
| Anti-htn Medication | -0.617 | 1.445 | -0.427 | 0.670 | -0.038 | [-3.449, 2.215] |
| PP X Age | 0.001 | 0.006 | 0.109 | 0.913 | 0.116 | [-0.010, 0.011] |
| PP X APOE | -0.102 | 0.091 | -1.120 | 0.265 | -0.096 | [-0.280, 0.076] |
| Age X APOE | 0.218 | 0.194 | 1.125 | 0.263 | 0.093 | [-0.162, 0.598] |
| **PP X Age X APOE** | 0.022 | 0.011 | 2.060 | **0.042** | 0.170 | [0.001, 0.043] |

**Supplemental Table S3:** Results from multiple linear regression models examining the relationship between pulse pressure (PP), age, APOE genotype, individual vascular risk factors, other potentially associated variables, and scores on each of the ten NIHTB-CB measures.

## Dimensional Change Card Sort Test

| Term | b | SE | t | p | β | 95% CI |
| --- | --- | --- | --- | --- | --- | --- |
| (Intercept) | 83.258 | 5.831 |  |  |  |  |
| PP | -0.051 | 0.058 | -0.885 | 0.377 | -0.106 | [-0.165, 0.062] |
| **Age** | -0.381 | 0.120 | -3.163 | **0.002** | -0.284 | [-0.617, -0.145] |
| **APOE** | -5.059 | 1.657 | -3.054 | **0.003** | -0.231 | [-8.306, -1.812] |
| **Education** | 0.924 | 0.313 | 2.954 | **0.004** | 0.206 | [0.311, 1.537] |
| Sex | -0.406 | 1.483 | -0.274 | 0.785 | -0.019 | [-3.312, 2.500] |
| Anti-htn Medication | -0.886 | 1.605 | -0.552 | 0.582 | -0.042 | [-4.032, 2.260] |
| BMI | -1.661 | 1.793 | -0.927 | 0.355 | -0.064 | [-5.175, 1.852] |
| Atrial Fibrillation | 2.143 | 2.967 | 0.722 | 0.471 | 0.050 | [-3.672, 7.958] |
| Hypercholesterolemia | 1.308 | 1.516 | 0.862 | 0.390 | 0.061 | [-1.664, 4.280] |
| Diabetes | -1.007 | 2.215 | -0.455 | 0.650 | -0.033 | [-5.347, 3.334] |
| PP X Age | -0.002 | 0.007 | -0.238 | 0.812 | 0.074 | [-0.015, 0.012] |
| PP X APOE | -0.044 | 0.098 | -0.452 | 0.652 | -0.033 | [-0.237, 0.148] |
| Age X APOE | 0.022 | 0.217 | 0.100 | 0.921 | 0.006 | [-0.403, 0.447] |
| PP X Age X APOE | 0.021 | 0.013 | 1.623 | 0.106 | 0.127 | [-0.004, 0.046] |

## Flanker Inhibitory Control and Attention Test

| Term | b | SE | t | p | β | 95% CI |
| --- | --- | --- | --- | --- | --- | --- |
| (Intercept) | 90.433 | 1.539 |  |  |  |  |
| **PP** | -0.137 | 0.048 | -2.877 | **0.004** | -0.230 | [-0.230, -0.044] |
| Age | -0.176 | 0.099 | -1.774 | 0.078 | -0.175 | [-0.371, 0.018] |
| APOE | -1.626 | 1.362 | -1.194 | 0.234 | -0.092 | [-4.295, 1.043] |
| Sex | 0.571 | 1.223 | 0.467 | 0.641 | 0.033 | [-1.827, 2.969] |
| Education | 0.846 | 0.258 | 3.281 | **0.001** | 0.231 | [0.341, 1.351] |
| Anti-htn Medication | 1.214 | 1.320 | 0.920 | 0.359 | 0.070 | [-1.373, 3.801] |
| BMI | 1.392 | 1.475 | 0.944 | 0.347 | 0.065 | [-1.499, 4.282] |
| Atrial Fibrillation | -0.164 | 2.440 | -0.067 | 0.947 | -0.005 | [-4.946, 4.618] |
| Hypercholesterolemia | -0.753 | 1.247 | -0.604 | 0.547 | -0.043 | [-3.197, 1.691] |
| Diabetes | -1.492 | 1.849 | -0.807 | 0.421 | -0.058 | [-5.116, 2.131] |
| PP X Age | 0.005 | 0.006 | 0.832 | 0.407 | 0.141 | [-0.006, 0.015] |
| PP X APOE | 0.053 | 0.081 | 0.658 | 0.511 | 0.049 | [-0.105, 0.211] |
| Age X APOE | -0.030 | 0.178 | -0.169 | 0.866 | -0.015 | [-0.380, 0.320] |
| PP X Age X APOE | 0.013 | 0.011 | 1.201 | 0.231 | 0.094 | [-0.008, 0.034] |

## Pattern Comparison Processing Speed Test

| Term | b | SE | t | p | β | 95% CI |
| --- | --- | --- | --- | --- | --- | --- |
| (Intercept) | 42.674 | 22.669 |  |  |  |  |
| PP | -0.114 | 0.082 | -1.397 | 0.164 | -0.099 | [-0.275, 0.046] |
| **Age** | -0.570 | 0.171 | -3.334 | **0.001** | -0.296 | [-0.906, -0.235] |
| **APOE** | -5.480 | 2.355 | -2.327 | **0.021** | -0.178 | [-10.096, -0.865] |
| Education | 0.387 | 0.200 | 1.934 | 0.055 | 0.135 | [-0.005, 0.779] |
| Sex | -0.321 | 2.084 | -0.154 | 0.878 | -0.011 | [-4.406, 3.763] |
| Anti-htn Medication | -1.629 | 2.280 | -0.714 | 0.476 | -0.054 | [-6.097, 2.840] |
| BMI | -0.540 | 2.545 | -0.212 | 0.832 | -0.015 | [-5.527, 4.448] |
| Atrial Fibrillation | 4.768 | 4.178 | 1.141 | 0.255 | 0.079 | [-3.421, 12.957] |
| Hypercholesterolemia | -2.167 | 2.149 | -1.008 | 0.315 | -0.072 | [-6.379, 2.045] |
| Diabetes | -3.492 | 3.217 | -1.085 | 0.279 | -0.080 | [-9.797, 2.814] |
| PP X Age | -0.002 | 0.010 | -0.192 | 0.848 | 0.033 | [-0.020, 0.017] |
| PP X APOE | 0.074 | 0.139 | 0.532 | 0.595 | 0.040 | [-0.198, 0.346] |
| Age X APOE | 0.047 | 0.308 | 0.153 | 0.878 | 0.011 | [-0.557, 0.651] |
| PP X Age X APOE | 0.016 | 0.018 | 0.860 | 0.391 | 0.067 | [-0.020, 0.052] |

## List Sorting Working Memory Test

| Term | b | SE | t | p | β | 95% CI |
| --- | --- | --- | --- | --- | --- | --- |
| (Intercept) | 2.506 | 19.038 |  |  |  |  |
| PP | -0.020 | 0.068 | -0.296 | 0.767 | -0.032 | [-0.154, 0.113] |
| **Age** | -0.631 | 0.143 | -4.414 | **<.001** | -0.413 | [-0.912, -0.351] |
| **APOE** | -9.046 | 1.961 | -4.614 | **<.001** | -0.314 | [-12.888, -5.203] |
| Education | 0.551 | 0.368 | 5.074 | .391 | 0.319 | [-0.522, 1.180] |
| Sex | -0.911 | 1.746 | -0.522 | 0.602 | -0.033 | [-4.333, 2.511] |
| Anti-htn Medication | -3.251 | 1.898 | -1.713 | 0.088 | -0.116 | [-6.972, 0.469] |
| BMI | -1.013 | 2.141 | -0.473 | 0.637 | -0.029 | [-5.209, 3.182] |
| Atrial Fibrillation | -0.583 | 3.618 | -0.161 | 0.872 | -0.010 | [-7.674, 6.509] |
| Hypercholesterolemia | -0.161 | 1.787 | -0.090 | 0.928 | -0.006 | [-3.664, 3.342] |
| Diabetes | 3.577 | 2.741 | 1.305 | 0.193 | 0.087 | [-1.795, 8.949] |
| PP X Age | -0.014 | 0.008 | -1.810 | 0.072 | 0.008 | [-0.030, 0.001] |
| PP X APOE | -0.017 | 0.116 | -0.147 | 0.884 | -0.010 | [-0.244, 0.210] |
| Age X APOE | -0.247 | 0.257 | -0.961 | 0.338 | -0.071 | [-0.749, 0.256] |
| **PP X Age X APOE** | 0.043 | 0.015 | 2.828 | **0.005** | 0.198 | [0.013, 0.073] |

## Picture Sequence Memory Test

| Term | b | SE | t | p | β | 95% CI |
| --- | --- | --- | --- | --- | --- | --- |
| (Intercept) | 83.799 | 7.105 |  |  |  |  |
| PP | 0.060 | 0.070 | 0.860 | 0.391 | 0.006 | [-0.077, 0.196] |
| **Age** | -0.664 | 0.147 | -4.514 | **<.001** | -0.341 | [-0.952, -0.376] |
| **APOE** | -4.444 | 2.022 | -2.198 | **0.029** | -0.161 | [-8.407, -0.481] |
| Education | 0.637 | 0.382 | 1.665 | 0.098 | 0.113 | [-0.113, 1.386] |
| **Sex** | 5.216 | 1.796 | 2.904 | **0.004** | 0.198 | [1.696, 8.737] |
| Anti-htn Medication | -3.603 | 1.940 | -1.857 | 0.065 | -0.136 | [-7.406, 0.200] |
| BMI | -2.544 | 2.150 | -1.183 | 0.238 | -0.079 | [-6.759, 1.670] |
| Atrial Fibrillation | 0.974 | 3.566 | 0.273 | 0.785 | 0.018 | [-6.014, 7.963] |
| Hypercholesterolemia | -1.454 | 1.840 | -0.790 | 0.430 | -0.055 | [-5.059, 2.152] |
| Diabetes | -1.535 | 2.694 | -0.570 | 0.569 | -0.040 | [-6.816, 3.745] |
| PP X Age | -0.008 | 0.008 | -0.980 | 0.329 | 0.073 | [-0.024, 0.008] |
| PP X APOE | -0.155 | 0.120 | -1.293 | 0.197 | -0.097 | [-0.390, 0.080] |
| Age X APOE | 0.290 | 0.263 | 1.106 | 0.270 | 0.079 | [-0.224, 0.805] |
| **PP X Age X APOE** | 0.044 | 0.016 | 2.808 | **0.006** | 0.210 | [0.013, 0.074] |

## Fluid Cognition Composite

| Term | b | SE | t | p | β | 95% CI |
| --- | --- | --- | --- | --- | --- | --- |
| (Intercept) | 72.909 | 6.631 |  |  |  |  |
| PP | -0.065 | 0.065 | -1.004 | 0.317 | -0.101 | [-0.192, 0.062] |
| **Age** | -0.685 | 0.138 | -4.949 | **<.001** | -0.422 | [-0.956, -0.414] |
| **APOE** | -6.712 | 1.881 | -3.569 | **<.001** | -0.255 | [-10.398, -3.026] |
| **Education** | 0.992 | 0.356 | 2.784 | **0.006** | 0.184 | [0.294, 1.691] |
| Sex | 1.085 | 1.685 | 0.644 | 0.520 | 0.043 | [-2.218, 4.388] |
| Anti-htn Medication | -2.303 | 1.807 | -1.275 | 0.204 | -0.091 | [-5.844, 1.238] |
| BMI | -1.309 | 2.025 | -0.646 | 0.519 | -0.042 | [-5.279, 2.661] |
| Atrial Fibrillation | 2.071 | 3.463 | 0.598 | 0.551 | 0.040 | [-4.716, 8.857] |
| Hypercholesterolemia | -1.011 | 1.712 | -0.591 | 0.555 | -0.040 | [-4.366, 2.344] |
| Diabetes | -1.588 | 2.598 | -0.611 | 0.542 | -0.041 | [-6.681, 3.505] |
| PP X Age | -0.004 | 0.008 | -0.529 | 0.597 | 0.097 | [-0.019, 0.011] |
| PP X APOE | -0.024 | 0.112 | -0.218 | 0.828 | -0.021 | [-0.243, 0.194] |
| Age X APOE | 0.046 | 0.245 | 0.189 | 0.850 | 0.008 | [-0.434, 0.527] |
| **PP X Age X APOE** | 0.037 | 0.014 | 2.586 | **0.011** | 0.188 | [0.009, 0.066] |

## Oral Reading Recognition Test

| Term | b | SE | t | p | β | 95% CI |
| --- | --- | --- | --- | --- | --- | --- |
| (Intercept) | 99.697 | 2.853 |  |  |  |  |
| PP | 0.007 | 0.028 | 0.248 | 0.805 | -0.023 | [-0.048, 0.062] |
| Age | 0.027 | 0.059 | 0.456 | 0.649 | 0.021 | [-0.089, 0.142] |
| APOE | -0.771 | 0.810 | -0.951 | 0.343 | -0.071 | [-2.359, 0.818] |
| **Education** | 0.727 | 0.153 | 4.752 | **<.001** | 0.329 | [0.427, 1.027] |
| Sex | -0.129 | 0.725 | -0.177 | 0.860 | -0.012 | [-1.551, 1.293] |
| Anti-htn Medication | -0.188 | 0.785 | -0.240 | 0.811 | -0.018 | [-1.728, 1.351] |
| BMI | -0.180 | 0.877 | -0.206 | 0.837 | -0.014 | [-1.899, 1.539] |
| Atrial Fibrillation | -0.016 | 1.452 | -0.011 | 0.991 | -0.001 | [-2.861, 2.829] |
| Hypercholesterolemia | -0.008 | 0.742 | -0.011 | 0.991 | -0.001 | [-1.462, 1.446] |
| **Diabetes** | -3.276 | 1.084 | -3.024 | **0.003** | -0.216 | [-5.400, -1.153] |
| PP X Age | 0.004 | 0.003 | 1.149 | 0.252 | 0.112 | [-0.003, 0.010] |
| PP X APOE | -0.040 | 0.048 | -0.838 | 0.403 | -0.062 | [-0.134, 0.054] |
| Age X APOE | -0.034 | 0.106 | -0.324 | 0.746 | -0.026 | [-0.242, 0.174] |
| PP X Age X APOE | 0.002 | 0.006 | 0.271 | 0.786 | 0.021 | [-0.011, 0.014] |

## Picture Vocabulary Test

| Term | b | SE | t | p | β | 95% CI |
| --- | --- | --- | --- | --- | --- | --- |
| (Intercept) | 97.219 | 4.882 | 19.914 | <.001 | 0.041 | [87.651, 106.788] |
| PP | 0.037 | 0.048 | 0.758 | 0.449 | -0.042 | [-0.058, 0.132] |
| **Age** | -0.308 | 0.101 | -3.055 | **0.003** | -0.203 | [-0.506, -0.110] |
| **APOE** | -3.049 | 1.387 | -2.199 | **0.029** | -0.160 | [-5.768, -0.331] |
| **Education** | 1.228 | 0.262 | 4.690 | **<.001** | 0.319 | [0.715, 1.741] |
| Sex | 2.077 | 1.241 | 1.673 | 0.096 | 0.115 | [-0.356, 4.510] |
| Anti-htn Medication | 1.263 | 1.344 | 0.940 | 0.349 | 0.069 | [-1.371, 3.897] |
| BMI | -0.044 | 1.501 | -0.029 | 0.977 | -0.002 | [-2.985, 2.898] |
| Atrial Fibrillation | 0.544 | 2.484 | 0.219 | 0.827 | 0.015 | [-4.325, 5.412] |
| Hypercholesterolemia | -1.578 | 1.269 | -1.243 | 0.215 | -0.086 | [-4.066, 0.910] |
| **Diabetes** | -4.107 | 1.854 | -2.215 | **0.028** | -0.155 | [-7.740, -0.473] |
| PP X Age | -0.006 | 0.006 | -1.044 | 0.298 | -0.040 | [-0.017, 0.005] |
| **PP X APOE** | -0.167 | 0.082 | -2.034 | **0.043** | -0.149 | [-0.328, -0.006] |
| Age X APOE | 0.217 | 0.182 | 1.196 | 0.233 | 0.091 | [-0.139, 0.573] |
| PP X Age X APOE | 0.009 | 0.011 | 0.818 | 0.414 | 0.062 | [-0.012, 0.030] |

## Crystallized Cognition Composite

| Term | b | SE | t | p | β | 95% CI |
| --- | --- | --- | --- | --- | --- | --- |
| (Intercept) | 97.971 | 3.758 | 26.067 | <.001 | 0.011 | [90.604, 105.337] |
| PP | 0.022 | 0.037 | 0.602 | 0.548 | -0.035 | [-0.051, 0.096] |
| **Age** | -0.157 | 0.078 | -2.020 | **0.045** | -0.142 | [-0.309, -0.005] |
| APOE | -2.101 | 1.068 | -1.968 | 0.051 | -0.143 | [-4.194, -0.008] |
| **Education** | 1.052 | 0.202 | 5.218 | **<.001** | 0.355 | [0.657, 1.447] |
| Sex | 0.985 | 0.956 | 1.030 | 0.304 | 0.071 | [-0.889, 2.858] |
| Anti-htn Medication | 0.612 | 1.035 | 0.591 | 0.555 | 0.043 | [-1.416, 2.640] |
| BMI | -0.134 | 1.155 | -0.116 | 0.908 | -0.008 | [-2.399, 2.130] |
| Atrial Fibrillation | 0.367 | 1.912 | 0.192 | 0.848 | 0.013 | [-3.381, 4.116] |
| Hypercholesterolemia | -0.929 | 0.977 | -0.950 | 0.343 | -0.066 | [-2.844, 0.987] |
| **Diabetes** | -3.980 | 1.427 | -2.788 | **0.006** | -0.195 | [-6.778, -1.182] |
| PP X Age | -0.001 | 0.004 | -0.255 | 0.799 | 0.011 | [-0.010, 0.007] |
| PP X APOE | -0.104 | 0.063 | -1.643 | 0.102 | -0.120 | [-0.228, 0.020] |
| Age X APOE | 0.093 | 0.140 | 0.665 | 0.507 | 0.050 | [-0.181, 0.367] |
| PP X Age X APOE | 0.005 | 0.008 | 0.576 | 0.566 | 0.044 | [-0.012, 0.021] |

## Cognitive Function Composite

| Term | b | SE | t | p | β | 95% CI |
| --- | --- | --- | --- | --- | --- | --- |
| (Intercept) | 83.282 | 5.130 | 16.235 | <.001 | -0.013 | [73.228, 93.336] |
| PP | -0.027 | 0.050 | -0.537 | 0.592 | -0.087 | [-0.125, 0.071] |
| **Age** | -0.489 | 0.107 | -4.563 | **<.001** | -0.378 | [-0.699, -0.279] |
| **APOE** | -5.179 | 1.455 | -3.560 | **<.001** | -0.254 | [-8.031, -2.328] |
| **Education** | 1.145 | 0.276 | 4.152 | **<.001** | 0.275 | [0.605, 1.686] |
| Sex | 1.216 | 1.304 | 0.932 | 0.352 | 0.062 | [-1.340, 3.771] |
| Anti-htn Medication | -1.078 | 1.398 | -0.771 | 0.441 | -0.055 | [-3.818, 1.661] |
| BMI | -0.875 | 1.567 | -0.558 | 0.577 | -0.036 | [-3.946, 2.197] |
| Atrial Fibrillation | 1.221 | 2.679 | 0.456 | 0.649 | 0.030 | [-4.030, 6.471] |
| Hypercholesterolemia | -1.186 | 1.324 | -0.895 | 0.372 | -0.060 | [-3.781, 1.410] |
| Diabetes | -2.912 | 2.010 | -1.449 | 0.149 | -0.098 | [-6.852, 1.028] |
| PP X Age | -0.003 | 0.006 | -0.578 | 0.564 | 0.079 | [-0.015, 0.008] |
| PP X APOE | -0.063 | 0.086 | -0.725 | 0.469 | -0.056 | [-0.232, 0.107] |
| Age X APOE | 0.074 | 0.190 | 0.390 | 0.697 | 0.024 | [-0.298, 0.446] |
| **PP X Age X APOE** | 0.026 | 0.011 | 2.325 | **0.021** | 0.169 | [0.004, 0.048] |
